# Control of thumb force using surface functional electrical stimulation and muscle load sharing

**DOI:** 10.1186/1743-0003-10-104

**Published:** 2013-10-09

**Authors:** Ard J Westerveld, Alfred C Schouten, Peter H Veltink, Herman van der Kooij

**Affiliations:** 1 Laboratory of Biomechanical Engineering, MIRA Institute for Biomedical Technology and Technical Medicine, University of Twente, 7500 AE Enschede, The Netherlands; 2 Department of Biomechanical Engineering, Delft University of Technology, 2628 CD Delft, The Netherlands

**Keywords:** FES, Load sharing, Muscle recruitment, Stroke, Rehabilitation, Force control, Thumb

## Abstract

**Background:**

Stroke survivors often have difficulties in manipulating objects with their affected hand. Thumb control plays an important role in object manipulation. Surface functional electrical stimulation (FES) can assist movement. We aim to control the 2D thumb force by predicting the sum of individual muscle forces, described by a sigmoidal muscle recruitment curve and a single force direction.

**Methods:**

Five able bodied subjects and five stroke subjects were strapped in a custom built setup. The forces perpendicular to the thumb in response to FES applied to three thumb muscles were measured. We evaluated the feasibility of using recruitment curve based force vector maps in predicting output forces. In addition, we developed a closed loop force controller. Load sharing between the three muscles was used to solve the redundancy problem having three actuators to control forces in two dimensions. The thumb force was controlled towards target forces of 0.5 *N* and 1.0 *N* in multiple directions within the individual’s thumb work space. Hereby, the possibilities to use these force vector maps and the load sharing approach in feed forward and feedback force control were explored.

**Results:**

The force vector prediction of the obtained model had small RMS errors with respect to the actual measured force vectors (0.22±0.17 *N* for the healthy subjects; 0.17±0.13 *N* for the stroke subjects). The stroke subjects showed a limited work range due to limited force production of the individual muscles. Performance of feed forward control without feedback, was better in healthy subjects than in stroke subjects. However, when feedback control was added performances were similar between the two groups. Feedback force control lead, especially for the stroke subjects, to a reduction in stationary errors, which improved performance.

**Conclusions:**

Thumb muscle responses to FES can be described by a single force direction and a sigmoidal recruitment curve. Force in desired direction can be generated through load sharing among redundant muscles. The force vector maps are subject specific and also suitable in feedforward and feedback control taking the individual’s available workspace into account. With feedback, more accurate control of muscle force can be achieved.

## Background

Stroke has become a major cause of morbidity and mortality in the western world. Incidence of stroke also increases in less developed countries as a result of changing life-styles [1]. Graying of society and improved health-care are likely to result in an increase of stroke survivors. Functional independence of stroke survivors is highly influenced by their ability to perform a successful grasp. In many activities of daily living, like drinking or opening a door, grasp and release is an essential part of the required movement.

Functional electrical stimulation (FES) of hand muscles can be helpful to train grasp and release in stroke subjects [2-4]. Depending on the ability of the individual patient, the assistance may be (selectively) increased or decreased in order to maximize the voluntary activity which is important in relearning movements [5,6].

Grasping comprises coordinated finger and thumb motion and controlled force exertion on the object to be held. As muscles initiate human movement, accurate control of muscle force is a prerequisite for movement control. For grasping tasks the fingers can be regarded as single degree of freedom (DoF) joints, since movement of the individual phalanges is coupled because of the under actuation of the finger. Furthermore, rotation along the flexion-extension axis of the finger is by far the most important movement for grasping and releasing objects. The thumb, however, requires a different approach as it moves along multiple axes. Controlling force and movement of the thumb will be most challenging and may serve as a model, which may be generalized/reduced to the single DoF case for the other fingers.

A healthy thumb is actuated in several directions by nine muscles in total [7,8]. However, not all nine muscles can be targeted properly with surface FES. Mainly, because of overlying muscles and nearby sensory nerves making stimulation uncomfortable. Therefore, only a small subset of thumb muscles is available for FES with surface electrodes. This limits the movements which can be controlled with FES. However, thumb movements relevant for grasping (mainly opposition) are feasible with surface electrodes.

Force distribution over multiple muscles is commonly applied in biomechanical modelling, solving actuator redundancy problems for a given task [9,10]. This load sharing approach might also be useful for activating a redundant muskuloskeletal system. In addition, by sharing the load over all available muscles we maximize the available range of force. However, to our knowledge, load sharing has not been applied to external activation of muscles with surface electrical stimulation. We will evaluate this possibility and expect this approach to result in accurate force control with a force distribution over the individual muscles optimized by minimizing the sum of squared recruitment over all muscles.

Recently, Lujan et al. [11] measured thumb forces evoked by three thumb muscles in healthy subjects and one spinal cord injured patient. Using the measured forces they trained an artificial neural network (ANN) for feed forward force control. They showed good control of the isometric thumb force in 2D. With the current study we aim at a more transparent approach: using linear combinations of estimated muscle force vectors instead of using a black-box ANN. This approach gives us the benefit of learning more of the underlying physiological system, by comparing combined muscle responses with individual muscle responses. In addition, it might allow for a more generally applicable approach, without the need of training an ANN.

The goal of the current study is twofold: 1) Is it possible to describe thumb muscle responses to FES by a sigmoidal muscle recruitment curve and a single direction of force? And if so, are these so called muscle force maps subject specific, suitable for stroke subjects and time-invariant? And 2) Are muscle force maps suitable for use in 2D thumb force control with FES applying load sharing? And if so, is feed forward control only sufficient and is the approach also suitable for stroke subjects?

## Methods

We will introduce the proposed generalized muscle force model for thumb force control and muscle load sharing first. Thereafter we will describe the experimental evaluation of this model in both healthy subjects and stroke subjects.

### Generalized muscle force model

We aimed at predicting muscle force resulting from FES by a relatively simple model. At a specific thumb posture we assumed that the force direction of each muscle, *ϕ*_*i*_, is constant and that a nonlinear sigmoidal relation exists between the stimulation amplitude and the generated muscle force.

(1) $$|{\vec{F}}_{i}(A_{i})|=\frac{p_{1i}}{1+e^{\frac{-(A_{i}-p_{2i})}{p_{3i}}}}-C,\;C=\frac{p_{1i}}{1+e^{\frac{p_{2i}}{p_{3i}}}}$$

**Figure 1:**
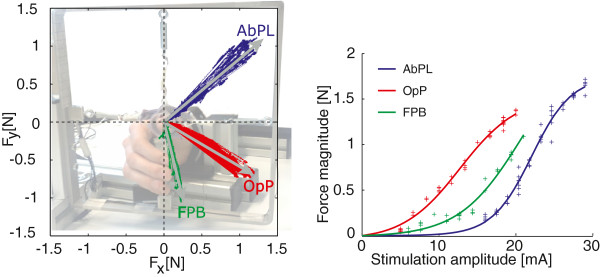
**Force vector map.** An example of the force vector map (direction (left) and magnitude (right)). The colored lines in the left pane show the measurement x- and y-forces for the abductor pollicis longus (AbPL), opponens pollicis (OpP) and the flexor pollicis brevis (FPB) muscles. The determined muscle force directions are indicated by the grey lines. The small variations indicate that the angles are relatively constant throughout the operating range. The force vector map in the left pane is shown on top of an overview of a custom built setup for restraining wrist movements and measurement of thumb forces with two pre loaded single axis force sensors. The fitted sigmoidal recruitment curves for the three thumb muscles and the individual measurement points (steady state of step responses at different amplitudes) are shown on the right.

In Eq. 1, $\left|{\vec{F}}_{i}(A_{i})\right|$ is the force magnitude of muscle *i* at stimulus amplitude *A*_*i*_; *p*_*i*1_ is related to the force saturation level, i.e. the maximal output force of that muscle, *p*_*i*2_ is related to the inflection point of the sigmoidal recruitment curve and *p*_*i*3_ is related to the horizontal scaling of the recruitment curve, i.e. the amplitude range. The latter term in Eq. 1 is an offset term, ensuring zero force if the amplitude is zero. The muscle force directions, together with the maximal force amplitudes for each muscle represents the force vector map for a system of multiple muscles, see Figure 1 for an example.

#### Feedforward thumb force model

We assumed a linear vector summation of the muscle forces acting around the same joint.

(2) $$\vec{F}=\overset{n}{\underset{i=1}{\sum }}x_{i}|{\vec{F}}_{\text{max},i}|\left[\;\;\begin{array}{c} \cos(\phi_{i})\\ \sin(\phi_{i}) \end{array}\;\right]$$

In Eq. 2, the predicted thumb force vector $\vec{F}$, is the vector sum of the individual muscle forces (*n*=3), modelled as a recruitment fraction, *x*_*i*_, of the maximal muscle force magnitudes, $\left|{\vec{F}}_{\text{max},i}\right|$.

The model of Eq. 2 was used to obtain the muscle stimulation levels given a desired thumb force. This inverse problem is redundant: three muscles can be stimulated to obtain a thumb force in two directions. In our (real-time) controller implementation, we addressed this redundancy problem by minimizing the squared muscle recruitment. Minimal summed force is a typical criterion also used in musculoskeletal modelling and load sharing studies [9,10]. The recruitment was modeled as a fraction of the maximal force, thus we obtained a bounded problem which can be formulated as minimizing the vector norm shown:

(3) $${\left|\left|F_{\text{max}}\vec{x}-{\vec{F}}_{r}\right|\right|}_{2}^{2}$$

In which ${\vec{F}}_{r}$ is the [2x1] column vector equal to the reference force and *F*_*max*_ is the [2x3] matrix containing the maximal *x* and *y* forces of each of the three muscles. $\vec{x}$ is the [3x1] column vector with individual muscle recruitment fractions. To take the bounds on *x* into account we reformulated the vector norm shown in 3 as the equation shown in Eq. 4.

(4) $$\underset{x\in [0,1]}{\text{argmin}}\;{\vec{x}}^{\text{T}}F_{\text{max}}^{\text{T}}F_{\text{max}}\vec{x}-2{\vec{F}}_{r}^{\text{T}}F_{\text{max}}\vec{x}+{\vec{F}}_{r}^{T}{\vec{F}}_{r}$$

Since the latter term is independent of *x*, the optimal recruitment, *x*, minimizing Eq. 4 can be written as a quadratic problem of the form as shown in Eq. 5, with $Q=F_{\text{max}}^{\text{T}}F_{\text{max}}$ and $\vec{c}=F_{\text{max}}^{T}{\vec{F}}_{r}$.

(5) $$\underset{x\in [0,1]}{\text{argmin}}\frac{1}{2}{\vec{x}}^{T}Q\vec{x}-{\vec{c}}^{T}\vec{x}$$

Finally the calculated reference forces for each muscle, $x_{i}|{\vec{F}}_{\text{max},i}|$, are converted to stimulation amplitudes by using the inverse of the sigmoidal recruitment (Eq. 1) curve shown in Eq. 6.

(6) $$A_{i}=-p_{3i}\ln\left(\frac{p_{1i}}{x_{i}|{\vec{F}}_{\text{max},i}|+C}-1\right)+p_{2i}$$

The combination of obtained stimulation amplitudes, *A*_*i*_, is the combination which theoretically would produce a force equal to the reference force, ${\vec{F}}_{r}$, or at least the force which is minimizing Eq. 3 when the system has reached its boundaries of operation. The constant *C* represents the offset term as introduced in Eq. 1.

### Model evaluation

#### Subjects

Five able bodied subjects (age 32 ± 13 years, 3 men) and five stroke subjects (age 55 ± 18, 4 men) were included for this study. Table 1 summarizes the characteristics for the individual stroke subjects. The study was in accordance with the declaration of Helsinki and was approved by the local medical ethics committee. All subjects gave written informed consent. During the experiments, the subjects were asked to relax their muscles, in order to avoid voluntary muscle activation.

**Table 1:** Characteristics of included stroke subjects

**Subject**	**Age**	**Sex**	**Affected side**	**Months**	**ARAT**
				**post-stroke**	
S1	50	M	L	44	52/57
S2	61	M	R	156	3/57
S3	69	M	L	45	24/57
S4	68	M	L	46	17/57
S5	26	F	L	58	2/57

The maximal obtainable Action Research Arm test (ARAT) score is 57 points (normal movement).

#### Experimental setup

Either the dominant arm (healthy subjects) or the affected arm (stroke subjects) was strapped in a custom built device. This setup was used to fixate the wrist and the hand in neutral pronosupination, and to measure the isometric thumb force in two directions perpendicular to the axis of the thumb. Forces were measured by two 45.3 *N* load cells (Futek, Irvine) preloaded with springs. See Figure 1.

A special built 3 channel asynchronous biphasic electrical stimulator (TIC Medizin, Dorsten, Germany) was used to apply the electrical stimulation pattern. Stimulation was applied at a constant frequency (30 Hz) and pulse width (150 *μs*). The amplitude could be controlled via custom built controllers within the stimulator’s range [0-30*mA*] in steps of 0.125*mA*. A single 50×50 *mm* anode was used together with 16×19 *mm* cathodes for each channel. Electrodes with similar size showed good results on both selectivity and comfort in a simulation study [12].

An EtherCAT I/O system (Beckhoff Automation GmbH, Verl, Germany) using Matlab/xPC (The Mathworks, Nattick, USA) as EtherCAT master device was used to control the stimulator parameters and to capture analog data from the force sensors.

#### Experimental protocol

##### Preparation

**Figure 2:**
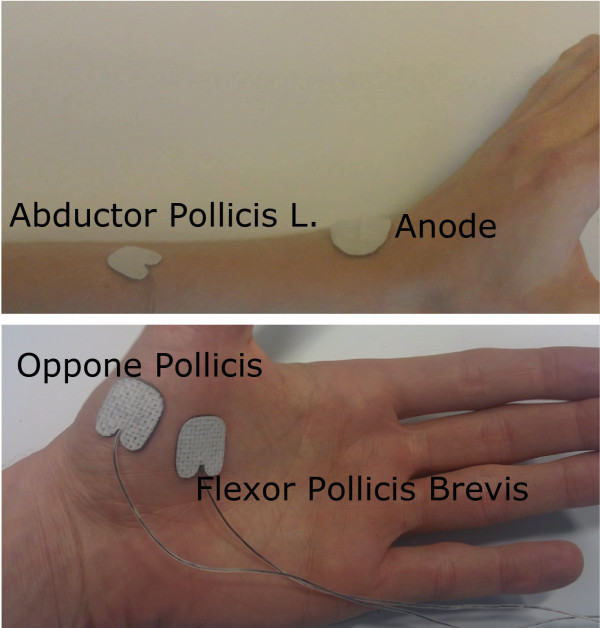
**Electrode placement.** Example of placement of electrode on (top) AbPL and placement of anode at the dorsum of the wrist and (bottom) above FPB muscle and OpP muscle. The AbPL electrodes was placed just medial of the radial bone, approximately 5 cm proximal to the wrist joint, the OpP electrode was placed laterally on the thenar, about 1/3 of the length of the first metacarpal bone, measured from the proximal side. The FPB electrode was placed at about half the length of the first metacarpal bone on the medial side of the thenar. Exact electrode locations were determined experimentally based on observed responses and subject comfort.

The Abductor pollicis longus (AbPL), Opponens pollicis (OpP) and Flexor pollicis brevis (FPB) muscles were selected for stimulation. We expected to move the thumb sufficiently in directions needed for grasp and release with these muscles. OpP opposes the thumb (pre-grasp), FPB moves the thumb inward (grasp) and AbPL moves the thumb up (release). Electrical stimulation was applied (30 *Hz*;150 *μs*) when electrodes were placed initially. The amplitude was increased to evaluate responses and subject comfort. Electrodes were located at the motor points based on exploration of the responses to electrical stimulation. See Figure 2 for an example of electrode placement.

##### Force vector map determination

The subject specific force map (see Figure 1 for an example) was determined in the isometric setup, with the thumb visually positioned at 30 degrees of abduction and 30 degrees of extension. The threshold and maximal stimulation amplitude for each muscle were determined first: we stimulated (30 *Hz*;150 *μs*) each muscle individually for 1 second, followed by 0.5 second without stimulation. Every 1.5 second the amplitude was increased by 1*mA*. When either a saturation in the force response was observed or the subject reported unpleasant discomfort, the stimulation was stopped.

The range between the threshold *minus* 1*mA* and the maximal amplitude was divided in ten equidistant stimulation levels for each muscle. We applied these 30 stimulations (10 amplitudes per muscle) randomly and measured the exerted thumb forces.

From this initialization measurement, we determined the force direction of each individual muscle and the recruitment curve relating muscle stimulation to exerted force. The recruitment curves were described with a sigmoidal function having three parameters, using Eq. 1. Parameter values were obtained with a least-squares fit, using the Levenberg-Marquardt algorithm [13]. See Figure 1 for an example of muscle recruitment curves and force directions. This force vector map indicates the ability to control the thumb force in different directions for a specific subject.

##### Individual muscle controllers

After determination of the force vector maps, the feedback controller gains were determined. Initial gains were obtained from an open loop Ziegler-Nichols step response procedure [14]. The step response reference pattern had the following sequence: [ 0.5 0.8 0.5 0.2 0.5]|*F*_*max*_|. The reference was held constant for three seconds at each specific level. Thus, excluding the steps at begin and end, this resulted in four step responses in total (two positive and two negative steps of step size 0.3|*F*_*max*_|). The signs of the negative step responses were inverted and then the average of all four step responses was used to determine the open loop gain, *K*_*o*_.

(7) $$K_{o}=\frac{X_{0}}{M_{u}}\frac{\tau }{\tau_{\text{dead}}}$$

In Eq. 7 the open loop gain, *K*_*o*_, is calculated from the normalized input magnitude, *X*_0_, the measured steady state output magnitude, *M*_*u*_, the time until the output responds, *τ*_*dead*_ and the time between the first response and the output reaching the steady state, *τ*. As suggested in [14], the proportional gain, *K*_*c*_, for each muscle was calculated as 90% of the open loop gain and the integration time for the PI-controller, *T*_*i*_, was set as 3.3 times *τ*_*dead*_. [14].

For every muscle and subject the inverse of the recruitment curve compensates the non-linear and subject and muscle specific recruitment. In this way the non-linear elements and maximal force levels are compensated within the control loop leading to a linear feedback controller between observed force error and reference force. Furthermore it is expected that range of control gains between the different muscles and different subjects is relatively small, since the muscle and subject specific recruitment curve transforms the outputs of the PI controllers (forces) into the required stimulation amplitudes.

After determining the initial gains for each muscle, in total four single muscle tests were done for each muscle to be able to analyze performances of the individual muscle controllers: 1) step response reference pattern with feedback control, 2) 0.5 Hz sinusoidal reference pattern with feedback controller, 3) step response reference pattern with a combination of feedforward and feedback control, and 4) 0.5 Hz sinusoidal reference pattern with a combination of feedforward and feedback control.

When oscillatory behaviour was observed during the first test, the proportional gain was lowered systematically and the test was repeated until good tracking of the reference was observed without severe oscillations. In some cases the integration time *T*_*i*_ was increased slightly for further fine tuning.

##### 2D thumb force targets

For evaluation of the 2D controllers, 5 second constant reference force targets were used. The targets were set at 0.5 *N* and 1.0 *N* in different directions within the workspace of the subject. Initially, directions were chosen at –90°, –60°, –30°, 0°, 30° and 60°. Angles outside the theoretical workspace of the subject were not measured. When less than four target directions were theoretically feasible, intermediate angles (15° step size) were also evaluated.

##### Feedforward thumb force control

The applicability of the thumb force model was evaluated first in an experiment based on feed forward control of the three muscles. In this experiment control was based on the measured muscle parameters and the thumb model described in Eq. 2. Based on the previously determined force map, target angles greater than the angle of the long abductor muscle or smaller. The experiment was repeated three times to explore the reproducibility of the methods. The target sequence was the same in each repetition. The sharing of the load was calculated by implementing Eq. 5 in a real-time quadratic programming (QP) problem solver using the online active set strategy [15].

##### Feedforward and Feedback thumb force control

**Figure 3:**
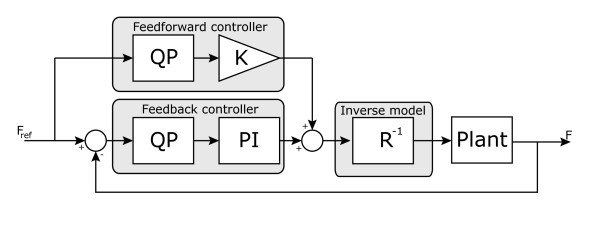
**Controller scheme.** Block diagram of feedforward and feedback thumb force controller. Stimulation for three individual muscles is calculated based on a reference force. Force distribution over the muscles is calculated by solving a QP problem as shown in 5 indicated by the 'QP’ blocks. These QP solvers take the previously determined force map and also boundaries on the recruitment into account. For clarity this is left out in the schematic. The bounds for the feedforward QP problem are [0,1]. The bounds for the feedback QP problem depend on the current activation of the muscle (from both feedforward and feedback path) and indicate the remainder of the operating range ([0,1]) and can thus also be negative when the specific muscle is already active. In the feedback path a PI controller was used for each individual muscle force. When using a combination of feedforward and feedback control, the feedforward path was reduced by a factor *K*=0.8 to prevent overshoot and let the feedback path compensate for the remainder. When evaluating the feedforward control performance without feedback, *K* was set to 1.

Control performance might be improved by adding error feedback. This was evaluated in a second set of control trials in which the feed forward control was extended with feedback error compensation. Force targets were the same as in the feed forward control experiments. The error vector between the reference force vector and the actual force vector was used as reference input for a second QP optimizer, which distributed the force error over the individual muscles. Note that due to feed forward muscle activation, forces can also be feedback controlled in the negative direction of the individual muscle axis. The calculated individual muscle force errors were fed back with the individual muscle controllers. A schematic overview of feedforward and feedback control paths is shown in Figure 3.

#### Performance analysis

RMS errors were calculated from the magnitude of the error vector between measured muscle force during the initialisation procedure and muscle force estimate based on the obtained parameters. In addition, the area of the theoretical work range resulting from the muscle force vectors obtained during the first initialization procedure was calculated and compared between subjects.

An important factor for the controllability is the rate of force change relative to the change of stimulation amplitude for a given muscle. This factor can be expressed by the maximal slope of the recruitment curve, calculated from the derivative of Eq. 1, for a give muscle, *i*:

(8) $${\text{slope}}_{\text{max},i}=\frac{p_{1}i}{4p_{3}i}$$

At the end of the session, we repeated the initialization procedure to check for possible changes in recruitment properties. In each repetition the sequence of applied amplitudes and selected muscles was kept the same. Time between subsequent initialization procedures was approximately 45 minutes. We estimated the correlation coefficients (Spearman’s *ρ*) between the measured forces and the forces predicted by the initially obtained model for each subjects. This gives an indication of both the prediction ability of the model and the repeatability of the method. To estimate effects of muscle fatigue we compared the force magnitudes in both initialization procedures and calculated the least squares slope, *m*, for each subject by:

(9) $$m=\frac{\sum |F_{\text{pre}}||F_{\text{post}}|}{\sum |F_{\text{pre}}{|}^{2}}$$

In which *F*_*pre*_ and *F*_*post*_, are the observed forces during the procedures at the beginning and the end of the session, respectively. The forces are summed over all applied input amplitudes during the initialization procedure. The slope, *m*, is an estimate of the ratio between initial force generation and final force generation for a given muscle.

Single muscle control performances were evaluated based on the sine tracking tasks. RMS errors between the actual and reference forces were calculated. The 2D controller performances were evaluated based on the stationary error of the responses. This stationary error was defined as the average magnitude of the force error vector during the last 10 percent of the in total 5 seconds lasting step response.

Due to the relatively small sample size, non-parametric statistics was applied. We used Mann Whitney U tests to statistically evaluate improvement with feedback control over feedforward control only and also to evaluate performance in stroke subjects with respect to healthy subjects.

## Results

### Force vector maps

**Figure 4:**
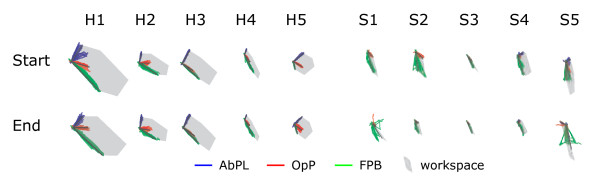
**Force vector map determination.** Force map data in subsequent force map measurements ('Start’ and 'End’ of experiment) for all (H)ealthy subjects and all (S)troke subjects. Grey arrows indicate maximal force for each muscle, obtained from the initialization procedure and the average movement direction. Axes were omitted for clarity, however the axes scaling was the same in all sub figures.

**Figure 5:**
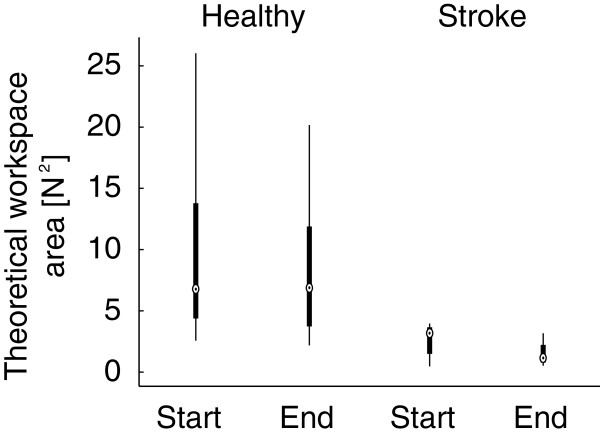
**Workspace areas.** Boxplots of theoretical workspace area for healthy subjects and stroke subjects. Workspaces calculated based on determined maximal muscle forces and muscle directions during the first initialization (Start) and the second initialization procedure (End).

Results of the initialization procedures for all subjects and all repetitions are summarized in Figure 4. Figure 5 shows the distribution of theoretical workspace area based on the determined muscle force maps for healthy subjects and stroke subjects. The workspace area was larger in healthy subjects, compared to stroke subjects: p=0.06 and p=0.02 for first and second initialization procedure respectively. RMS errors for the predicted force vectors were 0.10±0.02 *N*, 0.17±0.09 *N* and 0.19±0.11 *N* on average for the healthy subjects for AbPL, OpP and FPB, respectively. For the stroke subjects, the RMS errors were 0.66±0.12 *N* and 0.79±0.26 *N* for OpP and FPB, respectively. The AbPL muscle was only activated in S4 and S5, RMS errors were 0.14 *N* and 0.26 *N* for these subjects respectively. Maximal slopes of the recruitment curves in healthy subjects were 0.18±0.06 *N*/*A*, 0.17±0.06 *N*/*A* and 0.70±0.52 *N*/*A* for AbPL, OpP and FPB respectively. For the stroke subjects the maximal slopes were 0.09±0.06 *N*/*A* and 0.69±0.43 *N*/*A* for OpP and FPB respectively. The maximal slopes for the AbPL in subjects S4 and S5 were 0.07 *N*/*A* and 0.06 *N*/*A* respectively.

Correlations coefficients between predicted and measured forces are shown in Table 2 for both initialization procedures. The estimated force generation ratio’s between first and second initialization procedure in healthy subjects were 0.87±0.25, 0.93±0.10 and 0.97±0.06 for AbPL, OpP and FPB respectively. For the stroke subjects the ratio’s were estimated at 0.14±0.09 and 0.31±0.14 for OpP and FPB, respectively. For the AbPL muscle, the ratio’s were 0.35 and 0.29 for subjects S4 and S5 respectively.

**Table 2:** Force prediction

		**Healthy subjects**		**Stroke subjects**	
**Muscle**	**Procedure**	***F***_***x***_**correlation**	***F***_***y***_**correlation**	***F***_***x***_**correlation**	***F***_***y***_**correlation**
AbPL	initial	0.72±0.19	0.83±0.11	0.95±0.02	0.39±0.09
	final	0.61±0.22	0.77±0.14	0.84±0.22	-0.44±0.79
OpP	initial	0.80±0.13	0.73±0.31	0.51±0.56	0.79±0.09
	final	0.73±0.14	0.63±0.33	0.58±0.28	0.69±0.32
FPB	initial	0.88±0.06	0.92±0.06	0.47±0.20	0.82±0.24
	final	0.86±0.05	0.87±0.10	0.59±0.27	0.78±0.27

Correlations between predicted forces and measured forces during initialization procedures at start (initial) and end (final) of session.

### Force controller evaluation

#### Single muscle controllers

The averaged proportional gain over all healthy subjects was 0.22±0.28. For the stroke subjects the average proportional gain was 1.04±1.16, note that these values are dimensionless as the feedback controller has a force both as input and as output, since the inverse recruitment is placed after the controller. The average integral times were 0.56 ± 0.12*s* and 0.62 ± 0.45*s* for healthy subjects and stroke subjects respectively.

During the single muscle control experiments, some saturation effects (stimulation reaching predetermined maximal amplitude) were observed, leading to a non-linear feedback system. Disregarding the cases were this saturation occurred, the estimated controller gains were 0.17±0.12 and 0.57±0.12*s* on average for all subjects for proportional gain and integral time respectively.

**Figure 6:**
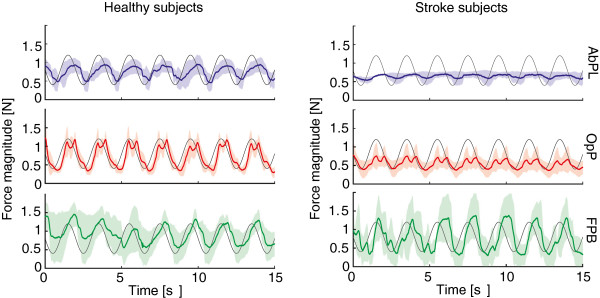
**Individual muscle control.** Sine (0.5 Hz) tracking results averaged over all healthy subjects (left) and over all stroke subjects (right). Results shown for the three selected muscles: Abductor Pollicis Longus (AbPL), Opponens Pollicis (OpP) and Flexor Pollicis Brevis (FPB) and for feedback control only. The mean over all subjects is shown by the solid line, shaded areas indicate the standard deviation. For AbPL only data for S4 and S5 is shown in (b), as in the other stroke subjects this muscle could not be activated.

Results of the sine tracking experiments for the individual muscle feedback controllers are shown in Figure 6. Results for healthy subjects and stroke subjects are shown separately. RMS tracking errors for the healthy subjects were 0.30±0.07 N, 0.29±0.06 N and 0.50±0.25 N for AbPL, OpP and FPB respectively. For the stroke subjects, RMS errors were similar: 0.31±0.03 N, 0.37±0.10 N and 0.52 ± 0.22 N for AbPL, OpP and FPB respectively. For subjects S1, S2 and S3 the AbPL muscle could not be targeted properly, therefore the AbPL tracking measurements were skipped for these subjects.

#### Combined muscle controllers

**Figure 7:**
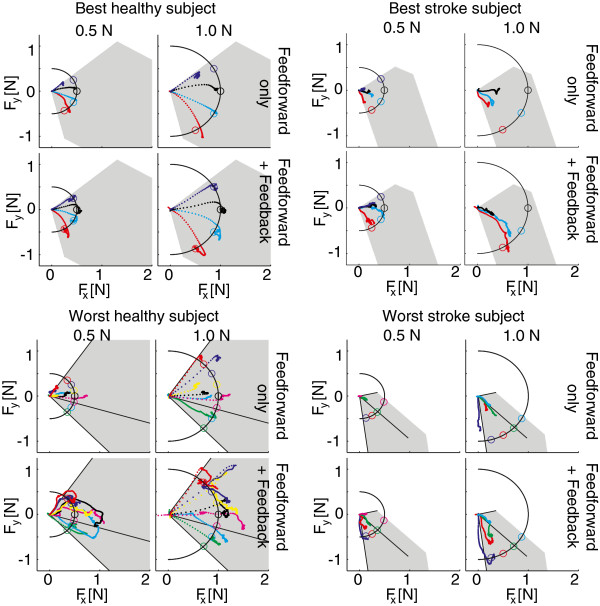
**2D force control.** Example of responses to the target set points for the best (H5; top-left) and the worst (H1; bottom-left) healthy subject and for the best (S4; top-right) and worst (S2; bottom-right) stroke subject. Top panes of each figure show results of solely feedforward control; bottom panes show results for feedforward and feedback control. 0.5 N targets (left) and 1.0 N targets (right) are shown separately for readability. The colored dotted lines show the measured force response to a target set point shown by the same colored circle in the plane perpendicular to the thumb. For every 100ms in the response a dot was plotted to give an indication of the speed of the force response.

**Figure 8:**
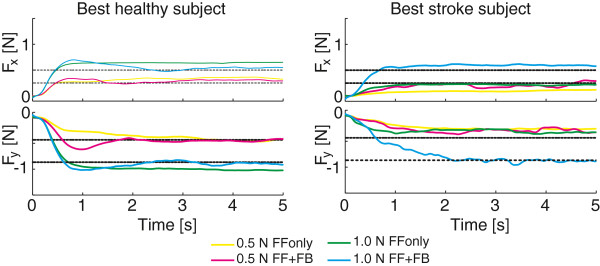
**Step responses of 2D force control.** Time series of responses to a step in target set point for healthy subject H5 (left) and stroke subject S4 (right). Top panes of each figure show forces in X direction; bottom panes show forces in Y direction.

**Figure 9:**
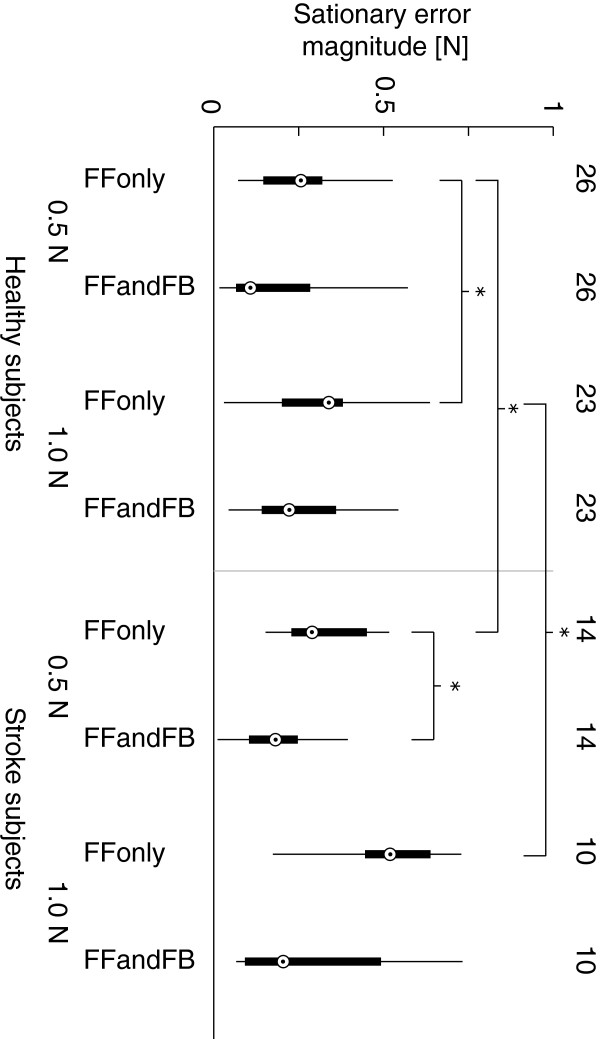
**Stationary errors in 2D force control.** Box plots of stationary errors in 2D force control trials. 0.5 N and 1.0 N targets are shown separately for feedforward control only and the combination feedforward and feedback control and for healthy subjects and stroke subjects. Numbers above the individual box plots indicate the total number of evaluated targets in that group, which was influenced by the workspace area of the individual subjects. Significant differences between groups were calculated by the non-parametric Mann-Whitney U test and indicated by asterisks.

2D step responses for the best (H5) and worst (H1) healthy subject and best (S4) and worst (S2) stroke subject are shown in Figure 7. Time series of stepresponses to a single 0.5 *N* target and a single 1.0 *N* target for H5 and S4 are shown in Figure 8. Responses over all subject are summarized in bar plots of stationary errors, shown in Figure 9. The stationary errors were averaged over all targets within a group. Results were grouped by control type, target magnitude and subject type. With feedback enabled, reduction in stationary errors was observed for the stroke subjects for the 0.5 N targets (p<0.1). Feedforward performance was less in stroke subjects, compared to the healthy subjects (p=0.05 and p<0.01 for the 0.5 N and 1.0 N targets respectively). The stationary errors were larger for the 0.5 N targets compared to the 1.0 N targets when normalized to the target forces (p<0.01) with feedforward control in healthy subjects. No significant differences in stationary errors were observed between the two target levels for the stroke subjects.

## Discussion

We showed the possibility to describe responses to electrical stimulation of individual thumb muscles as a force vector map with a single activation direction and a sigmoidal recruitment curve. As expected the variability between subjects is relatively large (Figure 4) due to anatomical differences. As a result, force maps always need to be determined for each individual subject. Within subject the results are repeatable, demonstrating the feasibility of our approach (Figure 4 and Table 2). Note that for subsequent sessions it is required to redo the initialization, since the response is highly dependent on exact electrode position [6]. However, in stroke subjects the AbPL muscle was difficult to target. In the subjects in which we were able to target the muscle initially, the responses during the second initialization procedure differed greatly from the initial procedure as indicated by the low correlation coefficients in Table 2 and in Figure 4. Therefore the AbPL muscle seems less reliable for use in 2D force control tasks compared to the other muscles.

The load sharing approach resulted in the muscle being pulled nicely towards the target force by the feedback controller. Since the error vector was used as input for the feedback load sharing, the appropriate ratio of muscle activations was calculated to generate force in the right direction. To our knowledge this load sharing approach is a novel application in electrically stimulated muscle. In our opinion this could be an appropriate solution to solve redundancy problems in activation of multi-dimensional muskuloskeletal systems with FES and simultaneously take the boundaries of the individual force sources into account. The variation of controller gains over different muscles and different subjects was low, which gives the possibility to use fixed values for these parameters when applying the methodology presented here. Either as a true fixed value of as a starting point for further fine tuning instead of the Ziegler-Nichols methods [14] which were currently used. Thereby further reducing the tunable parameters and setup time.

Performance of the 2D feedforward force controller was worse for the stroke subjects compared to the healthy subjects. For the stroke subjects, adding feedback terms reduced stationary errors. For the healthy subjects the differences between feedforward control only and combined with feedback control were small, see Figure 9. However, depending on the model accuracy of the individual muscle’s input-output relation, the feedback controller also reduced the control performance in certain cases. An example of this can be observed from Figure 7 where the feedback controller negatively influences the force direction for the 0.5 N targets. This is likely a result of a mismatch in the FPB model, causing the thumb being pulled in a more negative direction than needed. Therefore we recommend estimating model accuracy before starting the control trials, and redo the initialization if necessary.

### Limitations

We measured forces in two directions in a plane perpendicular to the thumb. Therefore we neglected the forces perpendicular to this plane. Due to this fact we might have made some errors in absolute force recordings. However, since we are using the same setup in both model identification and control, we expect that the influence of these non-measured forces on our performance observations are minimal.

Forces in unmeasured direction could have led to the relatively low observed forces compared to other studies [11]. However, we expect that these unmeasured forces were small. The stimulated muscles are responsible for thumb movement Therefore the force component in line width the thumb will be small compared to the perpendicular force components. A more likely cause is the fact that we aimed at selective activation with small electrodes leading to relatively low current densities and low muscle activation. Even though the observed forces and the evaluated targets of 0.5 N and 1.0 N are relatively low, they are sufficient for positioning the thumb for functional grasping of objects compared to the evaluated force levels during grasping in [16,17]. Recently, we have shown applicability of a similar approach during grasp and release of objects [18].

In all subjects, the FPB muscle showed a steep recruitment curve: when the stimulation came above threshold force increase was high for an increase in stimulation amplitude. This will have resulted in a bigger influence of FPB modelling errors on the output force errors. The steeper recruitment compared to other muscles is likely a result from differences in neural innervation. The FPB muscle is innervated from the recurrent branch of the median nerve which is very superficial before entering the FPB muscle. The OpP muscle is innervated by the same nerve branch, but laterally the branch runs less superficial [19]. The AbPL muscle is innervated by the posterior interosseus nerve which is also less superficial.

We reduced the experiment length by only testing specific points along the recruitment curve during the initialization phase. We did not specifically optimize this method of recruitment curve sampling. However, the results in pilot measurements where we compared our current approach with more dense sampling of the muscle recruitment resulted in only minor differences between the obtained recruitment curves. Recently, Schearer and colleagues [20] compared different methods of recruitment curve sampling extensively. Application of methods described there might further improve the accuracy of the obtained recruitment curves of individual muscles, which then could also improve the accuracy of the controllers.

The stroke subjects showed smaller workspaces compared to the healthy subjects (Figure 5). This is likely a result of non-use after stroke, which could have been overcome partially by additional muscle training prior to the experiment. However, since we only analyzed performance from the trials where the target force vector was within the theoretical workspace, this has not affected our current findings.

The ARAT scores of the stroke subjects had a broad range. Therefore the subjects cannot be considered as a homogeneous group. However, the emphasizes of the current approach lies on modelling subject specific recruitment relations. Therefore we did not observe lower stimulation responses related to lower ARAT scores. Furthermore, this is supported by the fact that the subjects with the best ARAT scores showed the smallest theoretical work range for the selected muscles.

### Physiological aspects

We expect the remainder of the variation to have a physiological cause. The most likely one is a non-linear additive relation between the individual muscle directions and recruitments. We expect that the linear addition of two individual force magnitudes to produce the desired combined force magnitude had the largest contribution to the remainder of the observed variability.

### Related work

Lujan and Crago [11] were able to control the thumb forces in two directions by using an artificial neural network. They also observed differences between the measured force of combined muscle activation and the sum of the individual components, which suggested a non linear additive relation. Lujan and Crago stimulated different muscles (Extensor Pollicis Longus, Abductor Pollicis Brevis and Adductor Pollicis). The evoked forces in that study are about five times higher than the forces which we found, possibly caused by higher stimulation frequencies (50 Hz compared to 30 Hz in our study) and the different set of stimulated muscles. This makes a good comparison between results difficult. Lujan and Crago only report 2D control RMS errors of one healthy subject and one spinal cord injured (SCI) patient, having implanted electrodes. The RMS error of the SCI patient was 0.89 *N*, which is very low compared to our results in stroke subjects when relating to the achieved force range. However implanted electrodes are known to produce higher muscle selectivity and more direct muscle activation, which makes this comparison unfair. The healthy subject they presented showed an RMS error of 2.65 *N*, which is (taking the factor 5 into account) within the same range as the stationary RMS errors we observed. However, we were able to obtain that similar performance without training and optimizing an artificial neural network but with a more transparent model consisting of only four parameters per muscle.

Schearer and colleges [21] recently published a single case study on controlling multiple degrees of freedom (in the shoulder) in a SCI subject with implanted electrodes using a feedforward controller. They also solved for redundancy by using a quadratic program and showed initial RMS errors of 5.29 *N*. As shoulder muscles are much stronger than thumb muscles, this value is again difficult to compare with our results. Given the range of their target forces (-18 *N* to 4.5 *N* in the *x* direction, -18 *N* to 22.5 *N* in the *y* direction and -9 *N* to 0 *N* in the *z* direction) one could say that the performance of their controller was slightly better than ours, which seems logical given the fact that the electrodes used by Schearer and colleagues were implanted. Therefore their stimulation was likely to result in more selective and accurate activation of individual muscles. In addition, Schearer *et al* suggest to improve the performance by adding a feedback controller, which is exactly what we did in the current study. We showed that adding the feedback path can indeed improve performance when the feedforward model is not accurate enough.

### Clinical implications

This study is a framework for evaluating multi-dimensional control of joints with electrical stimulation. To be clinically applicable in post-stroke rehabilitation, the method needs several extensions. First of all, we currently addressed only thumb muscles. For functional grasp and release training the finger muscles are of course equally important. However, compared to the thumb, those joints do not have the redundancy in actuation: mainly one extensor muscle and one superficial flexor muscle. Therefore the current method could easily be extended to the fingers, which we also evaluated recently [18].

When using additional electrodes for (selective) finger flexion and extension, the number of electrodes will increase quickly. Since, electrode placement is subject dependent and can be time consuming, the time required for setup will also increase rapidly. From a practical point of view, time can be gained with the application of electrode arrays and an approach to automatically search for proper electrode locations [22].

Finally, the relations between stimulation and movement and control of movement for grasp and release are also important. However proper force control is a fundamental prerequisite for proper control of movement. Therefore the current study can be seen as an intermediate step towards an approach for assisting grasp and release movements and next steps in our research will focus on directly mapping muscle activation to evoked movements.

Stroke subjects showed a limited workspace in our study. Since they did not have severe spasticity, it is likely that their muscle force have decreased dramatically due to long time non-use after their stroke. Therefore, we expect that results in more acute stroke subjects lie closer to those of the healthy subjects in the current experiment. However, this needs further evaluation and likely a subject specific approach will lead to the best results.

## Conclusion

The aim of this study was to evaluate the possibility to predict thumb muscle force responses to FES and to control thumb muscle force in 2D in both healthy and stroke subjects. For a single muscle, the static relation between muscle force and activation was described by a sigmoidal muscle recruitment curve and a single direction of force. Subsequently, load sharing was used to combine the activation of individual muscles to actively control thumb force in 2D.

From our results we can conclude that it is possible to describe the thumb muscle responses to FES by a single force direction and a sigmoidal recruitment curve. The large variations between subjects indicate that these force maps are highly subject specific, likely due to anatomical differences, requiring an individual approach. The relatively small variation within subjects demonstrates the feasibility and time-invariance of our approach. Effects of muscle fatigue were observed, especially in stroke patients, so the approach presented here is applicable mainly for short sessions (up to 30 minutes).

To our knowledge this is the first study applying a load sharing paradigm in controlling multiple muscles with surface FES in a multidimensional biomechanical system. The load sharing approach controlled the thumb towards the target forces in the 2D control experiments. With feedforward force control only, errors were larger in stroke subjects, compared to healthy subjects. However, with added feedback control, significant differences in control performance had disappeared. Therefore the methodology for multi-dimensional feedback force control presented here has potential applicability as part of post stroke rehabilitation techniques. Especially when applied earlier after stroke and muscles are stronger.

## Competing interests

The authors declare that they have no competing interests.

## Authors’ contributions

AW carried out the experiments, data analysis and drafting of the manuscript. AS, PV ad HK made substantial contributions to the study design, interpretation of the data and critical revision of the manuscript. All authors have read and approved the final manuscript.
